# Green analytical methods for quantification of a new combination therapy for amyotrophic lateral sclerosis: assessment by EPPI framework

**DOI:** 10.1038/s41598-026-66936-w

**Published:** 2026-08-20

**Authors:** Faten M. Aboukhalil, Essam F. Khamis, Mahmoud A. El-Sayed, Rasha M. Youssef, Amira F. El-Yazbi

**Affiliations:** Pharmaceutical Analytical Chemistry Department, Faculty of Pharmacy, Alexandria University, Alexandria, Egypt

**Keywords:** Ciprofloxacin, Celecoxib, Spectrophotometry, RP-HPLC, Plasma, EPPI framework, Sustainability and Amyotrophic lateral sclerosis, Biological techniques, Biotechnology, Chemistry

## Abstract

Amyotrophic lateral sclerosis is a progressive neurodegenerative disorder characterized by the degeneration of upper and lower motor neurons, leading to progressive muscle weakness, paralysis, and respiratory failure. The recently proposed combination therapy consisting of celecoxib and ciprofloxacin hydrochloride has emerged as a potential treatment for amyotrophic lateral sclerosis and other neurodegenerative disorders. The present study aimed to develop and validate simple, sensitive, environmentally friendly, and cost-effective analytical methods for the simultaneous determination of celecoxib and ciprofloxacin hydrochloride in bulk powders, laboratory-prepared tablets and plasma. Two ultraviolet spectrophotometric methods were established. The first method relies on direct absorbance measurements of ciprofloxacin hydrochloride at 318 nm and celecoxib at the isosbestic point at 266 nm, while the second one employs first-derivative ratio spectrophotometry for the selective determination of ciprofloxacin hydrochloride and celecoxib at 280 nm and 254 nm, respectively. Moreover, a reversed-phase high-performance liquid chromatographic method was developed using a C18 stationary phase, an optimized mobile phase composition and both diode array and fluorescence detectors, achieving efficient separation and accurate quantification of both analytes. The developed procedures were successfully applied to laboratory-prepared tablets and plasma following optimization of the sample preparation protocol to minimize matrix interference and to improve analytes recovery. Validation results showed excellent linearity over the investigated concentration ranges together with satisfactory accuracy, precision, selectivity, and sensitivity, confirming the reliability of the proposed methods for routine quality control and bioanalytical applications. The overall analytical performance and environmental sustainability of the developed methods were further assessed using the environmental performance and practicality Index framework, demonstrating their suitability as reliable and sustainable analytical approaches for the determination of celecoxib and ciprofloxacin hydrochloride.

## Introduction

**Fig. 1:**
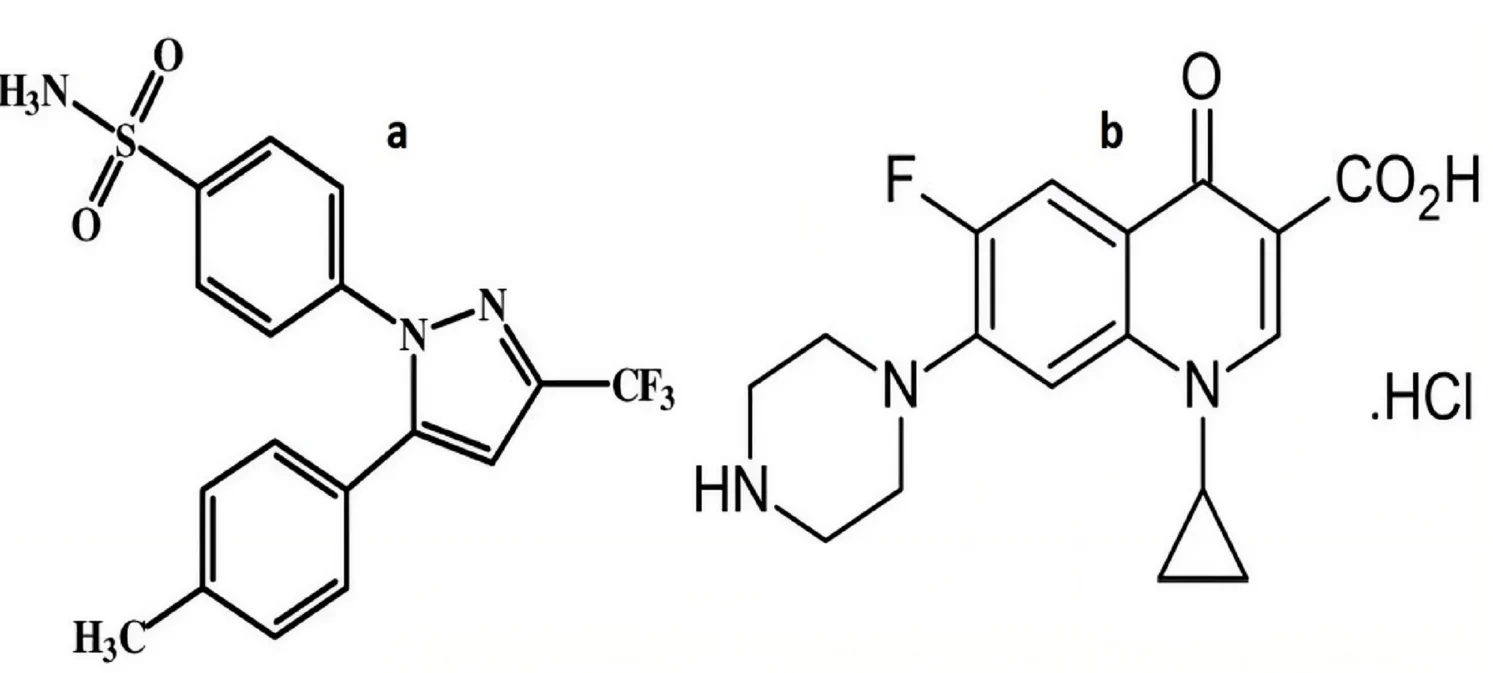
Chemical structure of Celecoxib (a) and Ciprofloxacin hydrochloride (b).

Celecoxib (CLX) (4-[5-(4-methylphenyl)-3-(trifluoromethyl)-1 H-pyrazol-1-yl]benzenesulfonamide)^1^(Fig. 1a) is an anti-inflammatory agent that acts by selectively inhibiting cyclooxygenase-2 (COX-2), a key enzyme involved in inflammatory processes. Unlike traditional NSAIDs, it exhibits a comparatively lower risk of inducing gastrointestinal complications such as ulceration and bleeding. Clinically, celecoxib is commonly prescribed to alleviate pain and inflammation associated with arthritic conditions. In addition to its established anti-inflammatory uses, celecoxib and other selective COX-2 inhibitors have gained interest for their possible roles in cancer prevention and therapy, leading to their evaluation in multiple clinical studies involving different malignancies. Therapeutically, celecoxib is approved for the symptomatic treatment of adult patients with osteoarthritis and rheumatoid arthritis^2^.

Ciprofloxacin hydrochloride (CPR) (1-cyclopropyl-6-fluoro-4-oxo-7-(piperazin-1-yl)-1,4-dihydroquinoline-3-carboxylic acid hydrochloride.)^3^(Fig. 1b) is a broad-spectrum antibacterial drug belonging to the fluoroquinolone group and is widely used in the management of numerous bacterial infections, including urinary tract and respiratory tract infections. It has received FDA approval for the treatment of a wide range of conditions such as urinary and gastrointestinal infections, sexually transmitted diseases, infections of the skin, bones, joints, and prostate, as well as typhoid fever, lower respiratory tract infections, salmonellosis, anthrax, and plague. Moreover, ciprofloxacin is considered a suitable therapeutic choice in cases involving mixed microbial infections or in patients who are at increased risk of infections caused by Gram-negative bacteria^4^. Prime C, an investigational combination of 34 mg CLX and 340 mg CPR, has emerged as a potential therapeutic candidate for the treatment of Amyotrophic lateral sclerosis (ALS) as well as Alzheimer’s disease^5,6^. This combination therapy has the advantages of maximizing therapeutic benefit by synergistic effect, enabling dose reduction, minimizing adverse effects, and preventing or delaying the onset of drug resistance^7^.

ALS is a catastrophic neurological disease that gradually dismantles the body’s ability to move while leaving cognition and awareness largely intact in many patients. It targets the motor neurons that act as the communication bridge between the brain, spinal cord, and skeletal muscles, and their progressive degeneration results in increasing muscle weakness, loss of voluntary movement, and severe physical disability^8^. What makes ALS particularly striking is the speed and irreversibility of its progression, transforming a previously independent individual into one who is fully dependent within a relatively short time. Understanding ALS today requires moving beyond the idea of a purely motor disorder and recognizing it as a systemic neurodegenerative condition with broad molecular and cellular implications. This perspective opens the door to innovative research strategies that focus on early detection, molecular profiling, and targeted intervention. By redefining ALS as a dynamic and multifaceted disease process, new opportunities arise for developing therapies that not only slow degeneration but also protect and potentially restore neuronal function^9^.

The literature reviews lack any of spectrophotometric or chromatographic methods for the simultaneous determination of CLX and CPR. The only literature review is spectrofluorometric method by Ahmed, A.B.,et al., 2025)^10^. So, it is of advantageous to develop the proposed methods which are novel, sensitive and economic ones with high performance and practicality.

The proposed methods are validated in accordance with ICH guidelines, assessing parameters such as linearity, precision, accuracy, limit of detection, limit of quantification and system suitability. The spectrophotometric methods offer a rapid, eco-friendly and economical alternative for routine quality control. Method I shows the direct determination of CPR at 318 nm and CLX at the isosbestic point at 266 nm. The direct determination of CLX is not feasible due to overlapping between the absorption spectra of CPR and CLX. So, the isosbestic point is effective to determine CLX within facilitating the resolution of overlapping spectra, enhances method selectivity, and improves the reliability of quantitative analysis in binary mixtures. Moreover, Method II is based on the first derivative ratio (FDR) of the absorption spectra which resolved overlapping problem with elimination of interference of background.

On the other hand, Reversed-phase high-performance liquid chromatography (RP- HPLC) represents a sensitive and selective technique suitable for complex matrices upon using fluorescence detector (FLD) and diode array detector (DAD). It is one of the most reliable and widely applied analytical techniques for the quantitative determination of drugs in plasma^11^. RP-HPLC method is highly applicable for the determination of the investigated drugs in dosage form and in plasma as result of its high separation efficiency, excellent sensitivity, and strong reproducibility when dealing with complex biological matrices. In this method, nonpolar stationary phases such as C18 columns combined with 80%v/v acetonitrile (ACN):20%v/v water enable effective retention and resolution of compounds with diverse physicochemical properties. This makes the technique particularly suitable for therapeutic drug monitoring, pharmacokinetic studies, and bioequivalence evaluations in addition to its ability to significantly reduce matrix interferences and to enhance analyte recovery. Furthermore, the sustainability and overall analytical effectiveness of the developed methods are assessed using the environmental performance and practicality index (EPPI), which is a recently introduced integrative comprehensive tool that avoids the limitation of other evaluation tools and incorporates green shipping practices (GSP), green analytical chemistry (GAC), and white analytical chemistry (WAC) principles. It is composed of environmental index (EI) which includes GSP and GAC principles for the evaluation of analytical method and performance and practicality index (PPI) which evaluate the practicality and performance of the methods^12^. EPPI provides a color scale (green for EI and purple for PPI) and a score from 1 to 100 for quantitative evaluation.

## Experimental

### Instrumentation

#### Spectrophotometer

Measurements were performed using UV-VIS spectrometer (Helios alpha, Unicam, England). All measurements were performed in 1-cm quartz cells. The instrument was connected to vision32 software. The absorbance data were processed and statistically analyzed using Microsoft Excel™ software.

#### HPLC

The HPLC Agilent 1200 series consisting of quaternary pump, autosampler, vacuum degasser; diode array G1315 C/D, multiple wavelength detector G1365 C/D and FLD detectors was used. Data acquisition and processing were performed using Agilent ChemStation software (Agilent Technologies, Santa Clara, CA, USA). Chromatographic separation was achieved on an Agilent Zorbax Eclipse Plus C18 analytical column (250 × 4.6 mm, 5 μm particle size). A Zorbax Eclipse Plus C18 guard column (12.5 × 4.6 mm, 5 μm particle size) was employed to protect the analytical column.

### Materials

CLX and CPR were obtained from Borg Pharmaceutical Industries, Area 3, 3rd Industrial zone, New Borg Elarab, Alexandria, Egypt. HPLC grade methanol and acetonitrile were products of Sigma- Aldrich Company.

### Preparation of standard stock solutions

2000 µg/mL stock solutions were prepared separately in methanol for CLX and in distilled water for CPR. Then 100 µg/mL working stock solutions of each drug were prepared separately from stock solutions to be used in spectrophotometric analysis. For RP-HPLC methods, 1000 µg/mL working stock solutions of each drug were prepared separately from stock solutions. The solvent which was used for dilution of the previously prepared working stock solutions was a mixture of 80%v/v methanol: 20% v/v water. 1000 µg/mL solution of metronidazole (MET) was prepared in HPLC methanol as internal standard (IS).

### General procedure of spectrophotometric methods

Accurate aliquots from working stock solutions were transferred separately into 5-mL volumetric flasks to obtain linearity range 5–30 µg/mL CLX and 10–60 µg/mL CPR for method I and 2–20 µg/mL and 3–20 µg/mL for method II for CLX and CPR, respectively.

#### Method I: Isosbestic spectrophotometric method

The prepared solutions were scanned from 200 to 400 nm then zero order absorption spectra for both investigated drugs were recorded after measuring the blank. CPR was determined using direct spectrophotometry at λ_max_ = 318 nm in presence of CLX without any interference, while CLX was determined at isosbestic point at 266 nm. This was done by construction of calibration curves at the selected wavelengths. Moreover, synthetic mixtures were measured at 318 nm for determining CPR content alone and at 266 nm for determining the total content of the investigated drugs in mixture. From the regression equations, the concentration of CPR alone and total content of CPR and CLX were calculated. The actual concentration of CLX alone was obtained by subtraction of CPR concentration from mixture concentration at isosbestic point.

#### Method II: First derivative ratio method

CLX was determined by dividing its zero order spectra by the spectrum of 3 µg/mL CPR as divisor without interference from CPR. In the same way, CPR was determined by dividing its zero order spectra by spectrum of 12 µg/mL CLX. The first derivative ratio spectra of each drug were obtained from the resulting absorption ratio of CLX and CPR. Then the calibration curves were constructed as a relation between first derivative ratio and concentrations at 254 nm for CLX and 280 nm for CPR.

### Chromatographic conditions of RP-HPLC method

The optimal separation system is based on using C-18 analytical column and C-18 guard column to protect the analytical column from damage. An isocratic system composed of 85%v/v ACN:15%v/v water was applied as mobile phase at 1mL /min flow rate. Membrane filter of 0.45 μm was used for double filtration of distilled water. The injection volume was 10 µL at ambient temperature (25^o^ C). The separation depended on two different detection system (DAD and FLD). DAD achieved high selectivity of the proposed method by determining CLX at 252 nm and CPR at 280 nm. Moreover, it ensured peak purity. On the other hand, FLD ensured high sensitivity of the analytical method to determine CLX at λ_ex_ = 245 nm and λ_em_ = 382 nm and CPR at λ_ex_ = 275 nm and λ_em_ = 445 nm.

### Analysis of the synthetic mixtures

Preparation of synthetic mixtures containing CLX and CPR was performed by serial dilution of stock solutions in solvent mixture to obtain concentrations ranges equivalent to 5–30 µg/mL, 2–20 µg/mL CLX and 10–60 µg/mL, 3–20 µg/mL CPR for method I and Method II, respectively. For RP-HPLC method, synthetic mixtures were prepared from CLX and CPR within linearity ranges (2–400 µg/mL CLX and 1–350 µg/mL CPR using DAD and 0.5–200 µg/mL CLX and 0.2–100 µg/mL CPR using FLD). Analysis of synthetic mixtures is a crucial to perform reproducibility and repeatability of the proposed methods.

### Analysis of laboratory-prepared tablets

Two laboratory-prepared tablets labeled to contain 340 mg CPR and 34 mg CLX were grinded and mixed well. Then a sample solution was prepared in solvent mixture to contain 1 mg/mL CPR and 0.1 mg/mL in 100-mL volumetric flask by the addition of 35-mL of the solvent mixture and the solution was sonicated for 20 min. followed by dilution to volume with the solvent mixture. This was followed by filtration using 0.45-µm membrane filter to obtain a clear solution. Then the working solutions were prepared by dilution with mobile phase for replicate measurements according to the optimal conditions.

### Analysis of spiked rat plasma

The animal study has been designed and the results have been reported in accordance with the ARRIVE Guidelines. All animal experiments were conducted according to an experimental protocol approved by the Institutional Animal Care and Use Committee at Alexandria University (Ethical Approval No. AU/06.2023.4.30.2.34) and in compliance with the Guide for the Care and Use of Laboratory Animals of the Institute for Laboratory Animal Research of the National Academy of Sciences, U.S.A. Male Sprague-Dawley rats were obtained from the animal facility at the Research and Innovation Hub at Alamein International University.

100 µg/mL working solutions of the investigated drugs were prepared separately from the stock solutions in the solvent mixture. In centrifuge tubes, aliquots of rat plasma (200µL) were spiked with different volumes of the working solution and a constant volume of IS. Then, a 2- mL volume of ACN was added to each test tube followed by vortex for 10 min and centrifugation at 2500 rpm for another 10 min. The supernatant solution was collected after repeating the previous step twice to be evaporated at vacuum concentrator. The obtained residues were reconstituted in 500µL mobile phase to get the calibration standards with the following concentrations 1, 2, 3, 4, 5, 10 and 20 µg/mL of CLX and CPR and 2 µg/mL of IS. Quality control solutions of CLX and CPR containing LQC (1 µg/mL), MQC (10 µg/mL) and HQC (20 µg/mL) representing low, medium and high-quality control solutions were prepared in rat plasma and were measured as five replicates according to FDA guide lines.

## Results and discussion

**Fig. 2:**
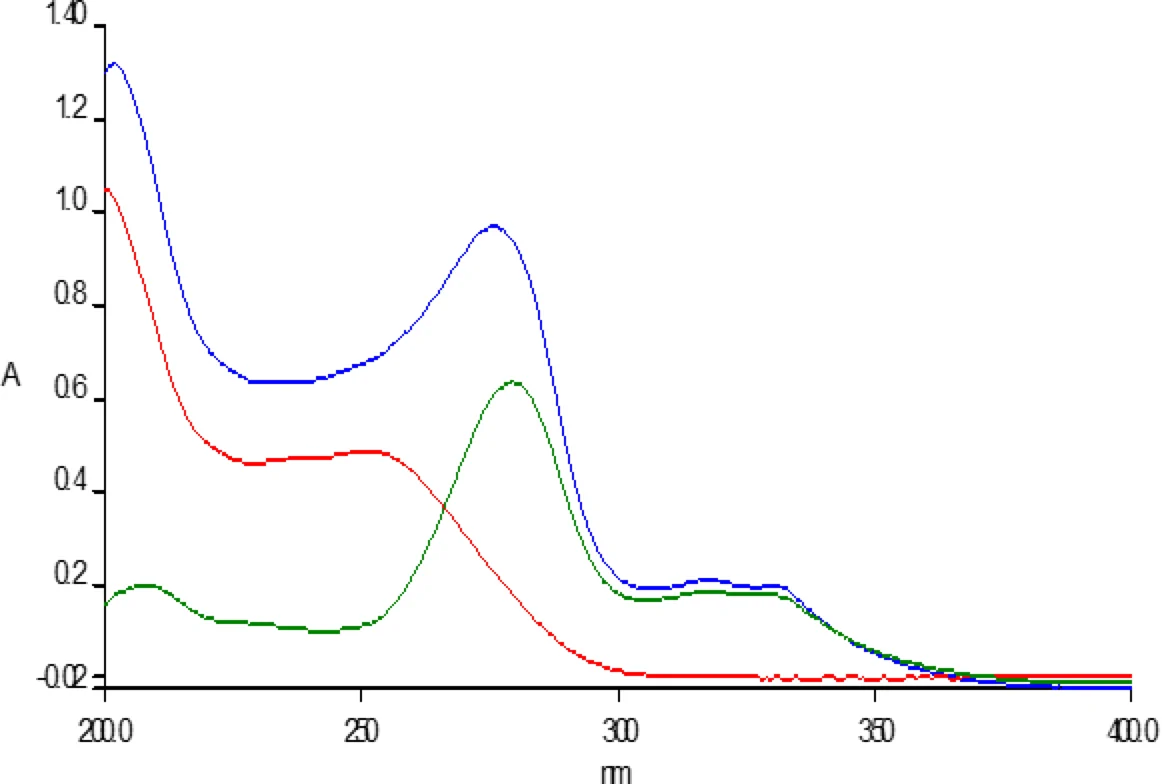
Zero Order absorption spectra of 10 µg/ mL CLX (red), 10 µg/ mL CPR (green) and 10 µg/ mL synthetic mixture (blue).

It is worth mentioning that it is the first time for simultaneous determination of this combination using either spectrophotometry or chromatographic techniques. The direct determination of CLX was not possible due to the overlapping of zero order absorption spectra of the investigated drugs as shown in Fig. 2.

### Spectrophotometric methods

#### Isosbestic spectrophotometric method

CPR can be directly determined at 318 nm without any interference from CLX, but there is an overlap between their spectra which makes the direct determination of CLX not feasible. Besides, the application of isosbestic point method (at 266 nm) makes it possible to determine both drugs under study without interference (Fig. 2).

#### Optimization of first derivative ratio method (FDR)

FDR is a spectrophotometric method that resolves overlapping problem of absorption spectra with elimination of interference of background. So, it provided an accurate analytical method for the analysis of the investigated binary mixture without the need for separation. Two parameters are need to be optimized, namely wavelength increment (∆ λ) and divisor concentration.

##### *Wavelength increment* (∆ λ)

**Fig. 3:**
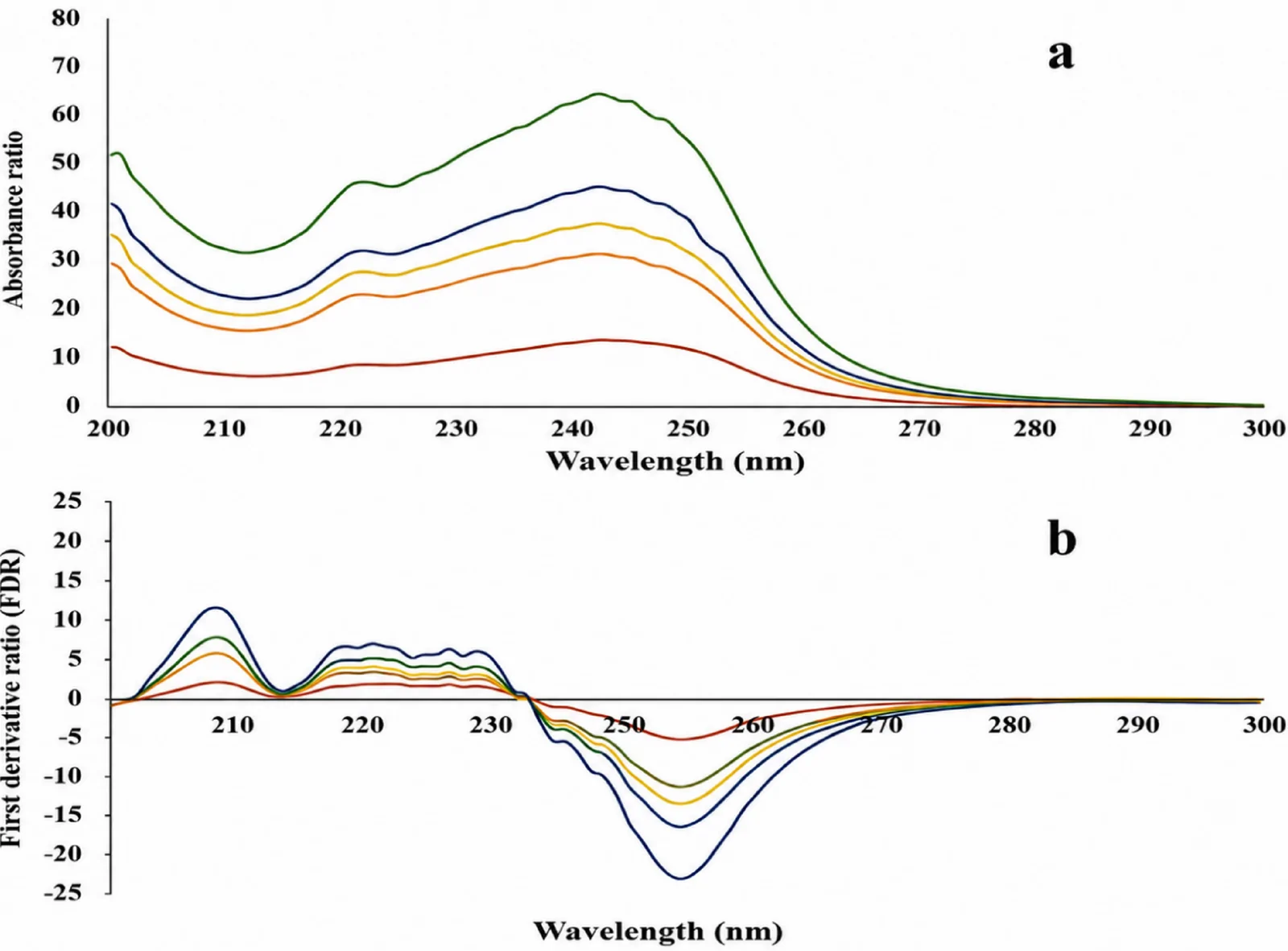
(**a**) Absorbance ratio and (**b**) first derivative ratio spectra of 5, 10, 12, 15, 20 µg/mL CLX using 3 µg/mL CPR as divisor.

**Fig. 4:**
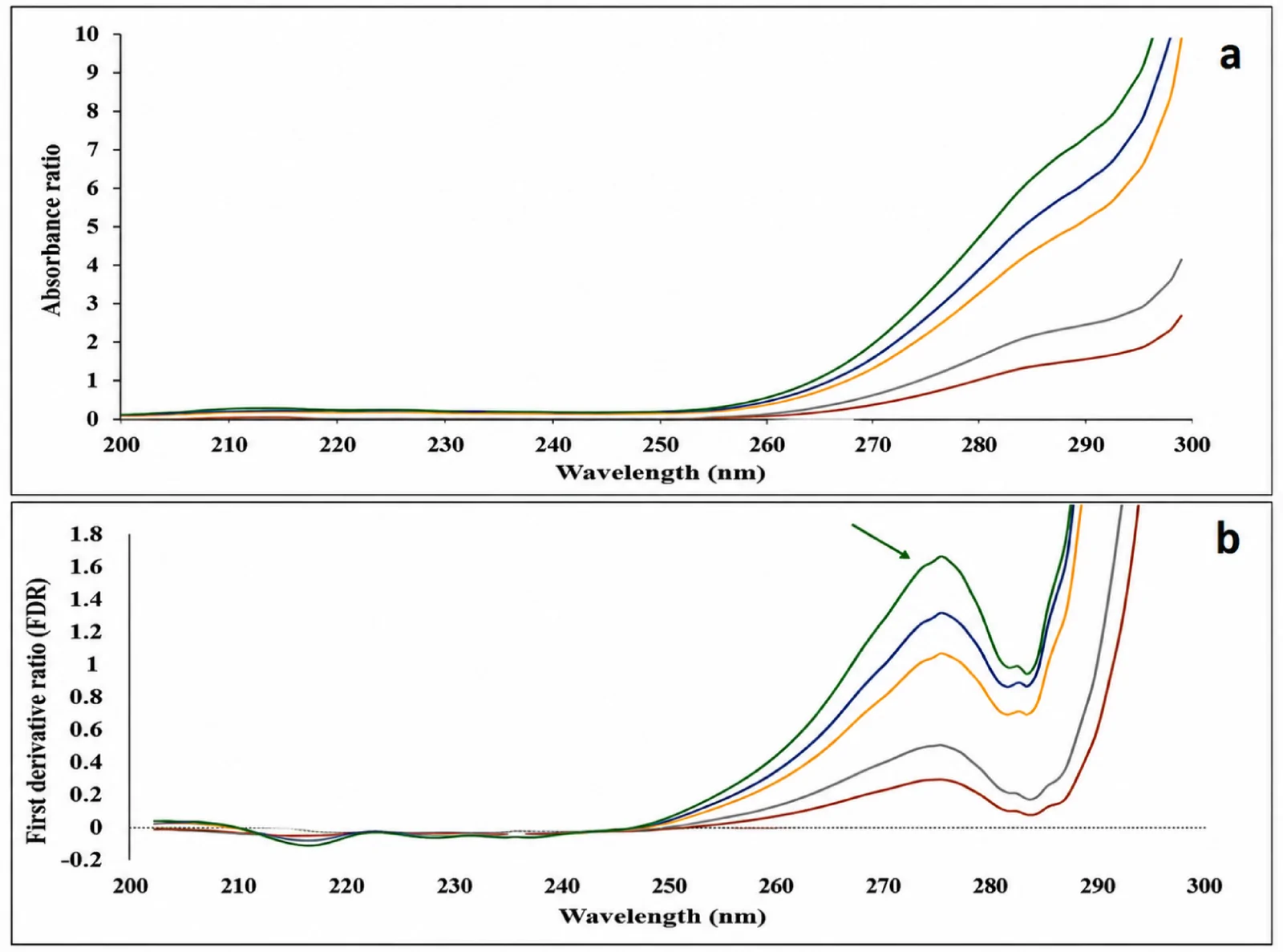
(**a**) Absorbance ratio and (**b**) first derivative ratio spectra of 3, 5, 10, 12, 15 µg/mL CPR using 12 µg/mL CLX as divisor.

To study the effect of ∆ λ on the derivative ratio spectra, different ∆ λs from 2 to 10 were studied. The best correlation coefficient and recoveries were obtained at ∆ λ 5 nm with small change in peak position. So, the most suitable wavelength increment for quantification of the investigated drugs is ∆ λ 5 (Figs. 3 and 4).

##### Divisor concentration

Different concentrations of divisor were studied as they affected the sensitivity of the method. The best repeatability and sensitivity were obtained by using 3 µg/mL CPR as divisor for the determination of CLX at 254 nm as shown in Fig. 3 µg/mL CLX as divisor for the determination of CPR at 280 nm without the need for separation steps (Fig. 4).

### RP-HPLC method

#### Optimization of chromatographic conditions

**Fig. 5:**
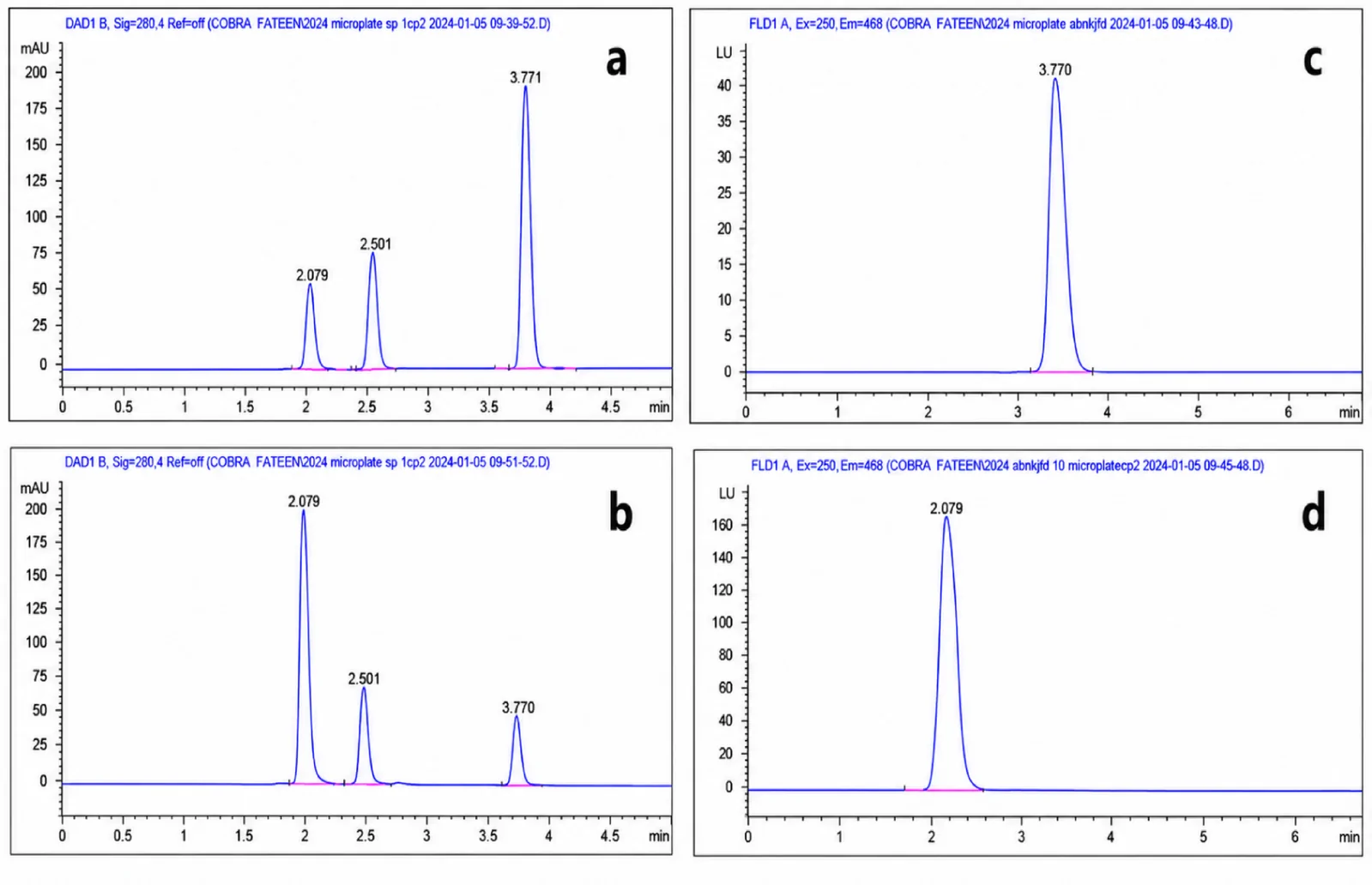
Typical HPLC chromatograms of a standard synthetic mixture containing 100: 30: 30 µg/mL of each of CPR, CLX and MET (IS) using DAD to determine (**a**) CLX at 252 nm, (**b**)CPR at 280 nm and using FLD to determine (**c**) CLX at λ_ex_ = 245 nm and λ_em_ = 382 nm and (**d**) CPR at λ_ex_ = 275 nm and λ_em_ = 445 nm.

Different buffers with variable pHs including phosphate and borate buffer have been examined but the obtained peaks were overlapping and broad. The most acceptable and sharp peaks were obtained upon using water with ACN as organic modifier. Also, organic modifier ratios have been tested from 20 to 80% v/v where the best retention time and capacity factors were reached upon using 80:20 v/v% ACN: water to obtain CPR at 2.08 min and CLX at 3.77 min (Fig. 5).

#### Selection of the internal standard (IS)

Several compounds with structural similarities to CPR, including ofloxacin, moxifloxacin, and levofloxacin, were investigated as potential internal standards. However, their chromatographic peaks showed significant overlap with the CPR peak. Ceftriaxone and metronidazole were also evaluated. MET was as ultimately selected as the internal standard owing to its suitable retention behavior, stability, absence in the studied matrix, and lack of interference with the analytes.

#### System suitability parameters (SSP)

According to the guidelines of ICH^13^, SSP are critical factors in RP-HPLC methods. They evaluate the separation time, column efficiency, resolution between peaks and the symmetry of the peaks. As demonstrated in Table 1, the results of SSP meet accepted criteria of ICH guidelines.

**Table 1:** System suitability parameters for the determination of binary mixture of CLX and CPR using RP-HPLC.

Parameters	CPR	CLX
t_R_ ± SD (min)^a^	2.08 ± 0.05	3.77 ± 0.08
Retention factors (k’)	3.16	6.54
Theoretical plates (N)	3073	10,106
USP tailing factor	1.25	1.20
Resolution (Rs)	2.81	8.40
Selectivity factor	1.26	1.64

^a^ Average of five determinations.

System suitability recommendations: k’> 2, R_s_ > 2, *N* > 2000, and T ≤ 2.

### Validation of the proposed methods

The ICH guidelines represent the most suitable reference for validation of the proposed methods^13^. Linearity is performed by construction of calibration curve which is a relationship between absorbance, FDR and peak area against concentration of CPR and CLX for method I, method II and RP-HPLC method, respectively. The results reveal that correlation coefficient values are more than or equal to 0.999 (Table 2). An increase in the F-values for equal degrees of freedom indicates a drop in the mean of squares due to residuals and an increase in the mean of squares due to regression. The higher the regression’s mean of squares, the more will be the regression line’s steepness. Regression lines with high F-values are, therefore significantly superior to those with lower ones. High values for both (r) and (F) are displayed by good regression lines^14^.

According to FDA guidelines^15^, the determination of lower limit of quantitation (LLOQ) has been applied by analysis of five replicates of small concentrations of each of investigated drug against blank of rat plasma (Table 3). LLOQ is the smallest concentration of drug with accepted % recovery (%R) and %RSD values. The evaluation of the precision within the same day (intra-day) and between different days (inter-day) and accuracy has been performed on three triplicates to show accepted %RSD and %Er (≤ 2%) as shown in Table 4. As for the biological fluid sample, five triplicates of QC samples have been measured to judge extraction recovery. The QC samples have % E_r_ and %RSD lower than 15% while LLOQ sample has values lower than 20% (Table 5). The specificity of the method has been ensured upon using DAD for the analysis of the investigated drugs in laboratory prepared tablets and upon spiking in rat plasma. The obtained peaks are of acceptable shapes without any interference. The stability study of the investigated drugs in biological fluid is so crucial. As demonstrated in Table 6, the stability of CPR and CLX in rat plasma was studied by the application of variable conditions and. Studying the effect of these different conditions on %R and %RSD of quality control samples show acceptable values ensuring the stability of the drug in biological samples (Table 6).

**Table 2:** Regression and statistical parameters for the determination of CLX and CPR using proposed methods.

Parameters	Spectrophotometric methods				RP-HPLC method			
	CPR		CLX		CPR		CLX	
	Method I	Method II	Method I	Method II	DAD	FLD	DAD	FLD
Linearity range (µg/mL)	10.0–60.0	3.0–20.0	5.0–30.0	2.0–20.0	1.0-350.0	0.2–100.0	2.0-400.0	0.5–200.0
LOQ (µg/mL)	2.12	0.68	0.85	0.67	0.94	0.15	1.37	0.27
LOD (µg/mL)	0.69	0.22	0.28	0.22	0.31	0.05	0.45	0.09
Intercept (a)	4.19 × 10^− 3^	4.00 × 10^− 3^	-4.96 × 10^− 3^	-6.77 × 10^− 1^	-2.87 × 10^1^	-1.01 × 10^2^	-2.16 × 10^1^	-1.38 × 10^1^
Slope (b)	1.73 × 10^− 2^	1.09 × 10^− 1^	3.61 × 10^− 2^	1.08	3.72 × 10^1^	2.15 × 10^2^	3.14 × 10^1^	2.28 × 10^1^
Correlation coefficient (r)	0.9999	0.9999	0.9999	0.9999	0.9999	0.9999	0.9999	0.9999
S_a_	2.84 × 10^− 3^	6.53 × 10^− 3^	2.72 × 10^− 3^	6.05 × 10^− 2^	1.78 × 10^1^	4.48 × 10^1^	2.67 × 10^1^	1.08 × 10^1^
S_b_	8.00 × 10^− 5^	5.32 × 10^− 4^	1.57 × 10^− 4^	4.94 × 10^− 3^	1.27 × 10^− 1^	1.15	1.29 × 10^− 1^	1.25 × 10^− 1^
S_b_^2^	6.30 × 10^− 9^	2.83 × 10^− 7^	2.47 × 10^− 8^	2.44 × 10^− 5^	1.62 × 10^− 2^	1.32	1.66 × 10^− 2^	1.56 × 10^− 2^
S_y/x_	3.67 × 10^− 3^	7.51 × 10^− 3^	3.08 × 10^− 3^	7.25 × 10^− 2^	3.87 × 10^1^	1.07 × 10^2^	4.87 × 10^1^	2.29 × 10^1^
F	47278.26	42167.80	52833.3	47945.5	85060.55	35018.34	59309.66	33563.01
Significance F	2.70 × 10^− 9^	3.40 × 10^− 9^	2.10 × 10^− 9^	4.79 × 10^− 9^	8.99 × 10^− 12^	8.27 × 10^− 11^	2.22 × 10^− 11^	9.19 × 10^− 11^

**Table 3:** Regression and statistical parameters for the simultaneous determination of CPR and CLX in spiked rat plasma using the proposed RP-HPLC method.

Parameter	Spiked plasma	
	CPR at 280 nm	CLX at 252 nm
Linearity range (µg/mL)	2.0–20.0	1.0–20.0
LLOQ (µg/mL)	2.0	1.0
Intercept (a)	-2.19 × 10^− 2^	5.63 × 10^− 2^
Slope (b)	2.71 × 10^− 1^	1.33 × 10^− 1^
Correlation coefficient (r)	0.9990	0.9991
S_a_	4.84 × 10^− 2^	2.21 × 10^− 2^
S_b_	4.27 × 10^− 3^	1.98 × 10^− 3^
S_b_^2^	1.83 × 10^− 5^	3.93 × 10^− 6^
S_y/x_	6.72 × 10^− 2^	9.57 × 10^− 3^
F	4021.15	4545.54
Significance F	3.70 × 10^− 7^	2.90 × 10^− 7^

**Table 4:** Intra-day and inter-day precision and accuracy for the determination of binary mixtures of CLX and CPR using the proposed methods.

Concentration (µg / mL)		Proposed Methods		Mean % Recovery ± SD (%)		%RSD		% E_*r*_	
CPR	CLX			CPR	CLX	CPR	CLX	CPR	CLX
(a) Accuracy and intra-day precision									
10	5	**Spectrophotometry**	**Method I**	100.90 ± 0.87	100.96 ± 1.08	0.86	1.07	0.90	0.96
20	30			99.53 ± 1.21	98.56 ± 0.54	1.22	0.55	-0.47	-1.44
60	10			99.27 ± 0.82	100.40 ± 1.05	0.83	1.05	-0.73	0.40
3	20		**Method II**	100.65 ± 1.34	99.74 ± 0.46	1.33	0.46	0.65	-0.26
10	10			101.20 ± 0.97	101.43 ± 0.98	0.96	0.97	1.20	1.43
15	5			98.45 ± 0.33	100.41 ± 1.25	0.34	1.25	-1.55	0.41
10	5	**RP-HPLC**	**DAD**	101.20 ± 1.01	99.64 ± 1.08	0.99	1.08	1.2	-0.36
450	400			98.87 ± 0.98	99.48 ± 1.83	0.99	1.84	-1.13	0.52
350	200			100.95 ± 0.57	98.87 ± 0.66	0.57	0.67	0.95	-1.13
1	1		**FLD**	99.12 ± 0.27	100.47 ± 0.44	0.27	0.44	-0.88	0.47
50	200			101.10 ± 1.40	101.03 ± 1.33	1.38	1.32	1.10	1.03
100	100			100.66 ± 0.36	101.41 ± 0.15	0.36	0.15	0.66	1.41
**(b) Accuracy and inter-day precision**									
10	5	**Spectrophotometry**	**Method I**	99.08 ± 0.67	99.96 ± 0.18	0.68	0.18	-0.92	-0.04
20	30			101.47 ± 0.65	99.06 ± 0.44	0.64	0.44	1.47	-0.94
60	10			100.22 ± 0.97	101.50 ± 0.69	0.97	0.68	0.22	1.50
3	20		**Method II**	101.09 ± 1.17	98.78 ± 1.25	1.16	1.27	1.09	-1.22
10	10			98.78 ± 0.77	100.56 ± 0.61	0.78	0.61	-1.22	0.56
15	5			98.15 ± 1.11	101.41 ± 0.35	1.13	0.35	-1.85	1.41
10	5	**RP-HPLC**	**DAD**	99.35 ± 0.33	100.64 ± 0.62	0.33	0.62	-0.65	0.64
450	400			99.47 ± 1.11	101.56 ± 0.47	1.12	0.46	-0.53	1.56
350	200			101.00 ± 1.02	99.54 ± 0.11	1.01	0.11	1.00	-0.46
1	1		**FLD**	100.14 ± 1.27	99.47 ± 0.17	1.27	0.17	0.14	-0.53
50	200			99.14 ± 0.39	100.17 ± 0.47	0.39	0.47	-0.86	0.17
100	100			101.60 ± 1.27	100.55 ± 0.65	1.25	0.65	1.60	0.55

**Table 5:** Intra-day and inter-day precision and accuracy for the determination of binary mixtures of CLX and CPR spiked in rat plasma using the proposed RP-HPLC methods.

Concentration (µg / mL)		Mean % Recovery ± SD (%)		%RSD		% E_*r*_	
CPR	CLX	CPR at 280 nm	CLX at 252 nm	CPR	CLX	CPR	CLX
(a) Accuracy and intra-day precision							
2	4	108.31 ± 11.86	96.03 ± 9.45	10.95	9.84	8.31	-3.97
4	1	95.42 ± 7.23	110.00 ± 7.04	7.57	6.4	-4.58	10.00
10	10	106.28 ± 10.30	105.36 ± 11.68	9.69	11.08	6.28	5.36
20	20	90.25 ± 11.98	104.96 ± 10.23	13.27	9.75	-9.75	4.96
**(b) Accuracy and inter-day precision**							
2	4	96.22 ± 13.39	98.5 ± 6.35	13.91	6.45	-3.78	-1.50
4	1	103.39 ± 12.98	109.34 ± 2.31	12.55	2.11	3.39	9.34
10	10	92.09 ± 7.98	104.97 ± 3.28	8.66	3.12	-7.91	4.97
20	20	95.31 ± 4.01	114.31 ± 11.20	4.21	9.79	-4.69	14.31

**Table 6:** Stability studies for CPR and CLX in spiked rat plasma using the proposed RP-HPLC method.

Stability	Storage conditions	Conc.	Average %recovery ± %RSD^a^	
			Rat plasma	
			CPR	CLX
Freeze and thaw	3 cycles at -20 °C for 1 h	LQC	90.85 ± 3.59	96.65 ± 11.25
		HQC	106.34 ± 2.17	88.83 ± 8.96
Short term	At 25 °C for 8 h	LQC	114.91 ± 9.40	113.34 ± 9.36
		HQC	90.15 ± 4.31	96.98 ± 5.24
Post-preparative	At 4 °C for 24 h	LQC	85.70 ± 8.70	104.00 ± 9.31
		HQC	94.09 ± 7.99	97.77 ± 10.53
Long term	At-20 °C for 10 days	LQC	110.28 ± 5.36	95.47 ± 3.25
		HQC	86.06 ± 11.23	106.25 ± 2.13

^a^ Percentage of recoveries average ± relative standard determination of three replicates (*n* = 3).

### Application of chromatographic method

**Fig. 6:**
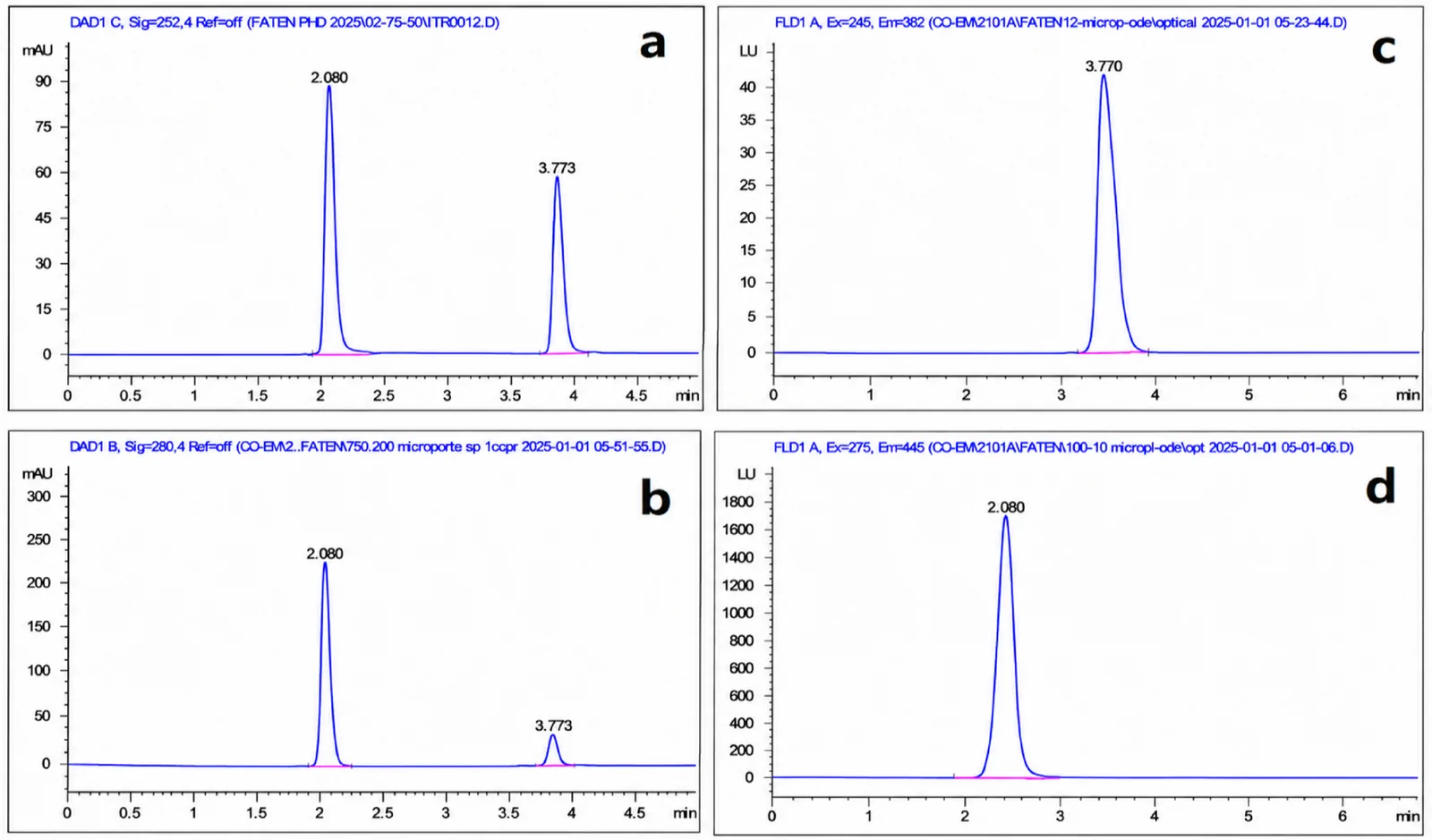
Typical HPLC chromatograms of 100 µg/mL: 10 µg/mL of each of CPR and CLX in laboratory prepared tablet using DAD to determine (**a**) CLX at 252 nm, (**b**)CPR at 280 nm and using FLD to determine (**c**) CLX at λ_ex_ = 245 nm and λ_em_ = 382 nm and (**d**) CPR at λ_ex_ = 275 nm and λ_em_ = 445 nm.

The proposed chromatographic method was applicable for the determination of investigated drugs in laboratory prepared tablets with accepted values of %R and %E_r_ as demonstrated in Fig. 6. Moreover, the method was also applicable for the analysis of drugs in spiked rat plasma. This makes the technique particularly suitable for therapeutic drug monitoring, pharmacokinetic studies, and bioequivalence evaluations.

### Analysis of spiked plasma

**Fig. 7:**
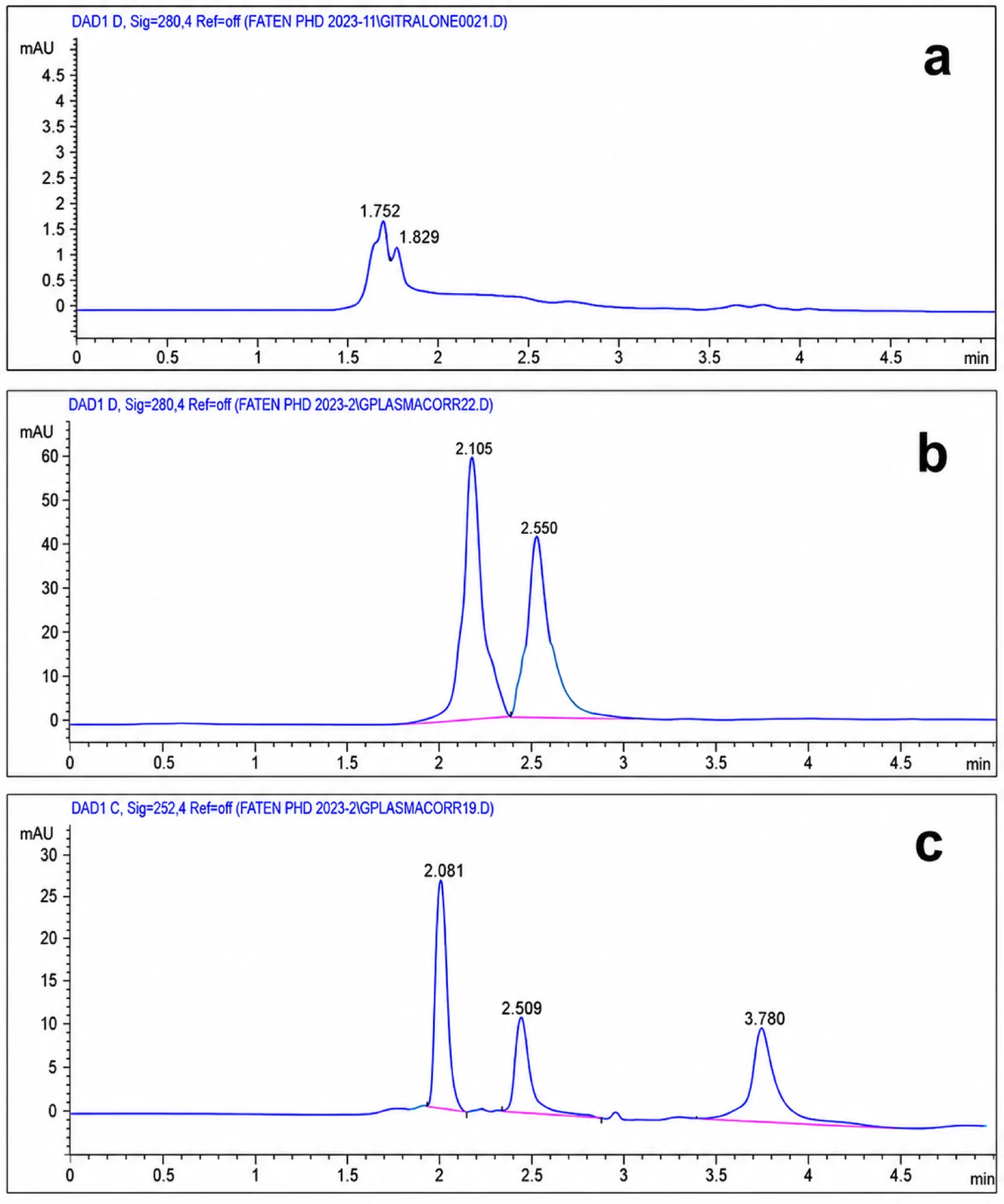
Typical HPLC chromatogram of (**a**) blank plasma, chromatogram of 20 µg/mL CPR: 2 µg/mL CLX spiked in plasma in presence of 2 µg/mL MET (IS) using (**b**) DAD at 280 nm and (**c**) DAD at 252 nm.

The comparison of the proposed HPLC method with the reported fluorometric one reveals that the former offers several important advantages when analyzing drugs in complex biological matrices (plasma, serum, and urine) compared with spectrofluorimetric methods. HPLC physically separates the drug from endogenous components and reduces matrix interference, which is a major limitation in spectrofluorimetry where many biological constituents may fluoresce. It has higher selectivity and specificity and provides better accuracy and precision in complex matrices and more reliable quantification in biological fluids with variable composition. In HPLC, separation step minimizes overlapping signals. Moreover, it enables simultaneous determination of the investigated drugs in the same run (Fig. 7), while spectrofluorimetry usually measures one fluorescent species at a time. Thus, while spectrofluorimetry is simple and sensitive for relatively clean samples, HPLC is superior for biological fluids due to its high selectivity, reliability, and regulatory acceptance in drug bioanalysis.

### Comparison with reported method

Only one synchronous spectrofluorometric method is reported on simultaneous analysis of CLX and CPR combination therapy (Ahmed, A.B., et al., 2025)^10^. The proposed spectrophotometric method is simpler, easier to use, more applicable and more economic than the reported one. Also, the proposed RP-HPLC has several advantages over the reported fluorometric method as it has superior separation and selectivity, reduced effect of biological matrix, multi-component analysis in the same run and the variety in the detection system (Table 7). So, RP-HPLC method is more applicable than fluorimetry in analysis of complex matrices (as rat plasma).

### Greenness assessment

**Fig. 8:**
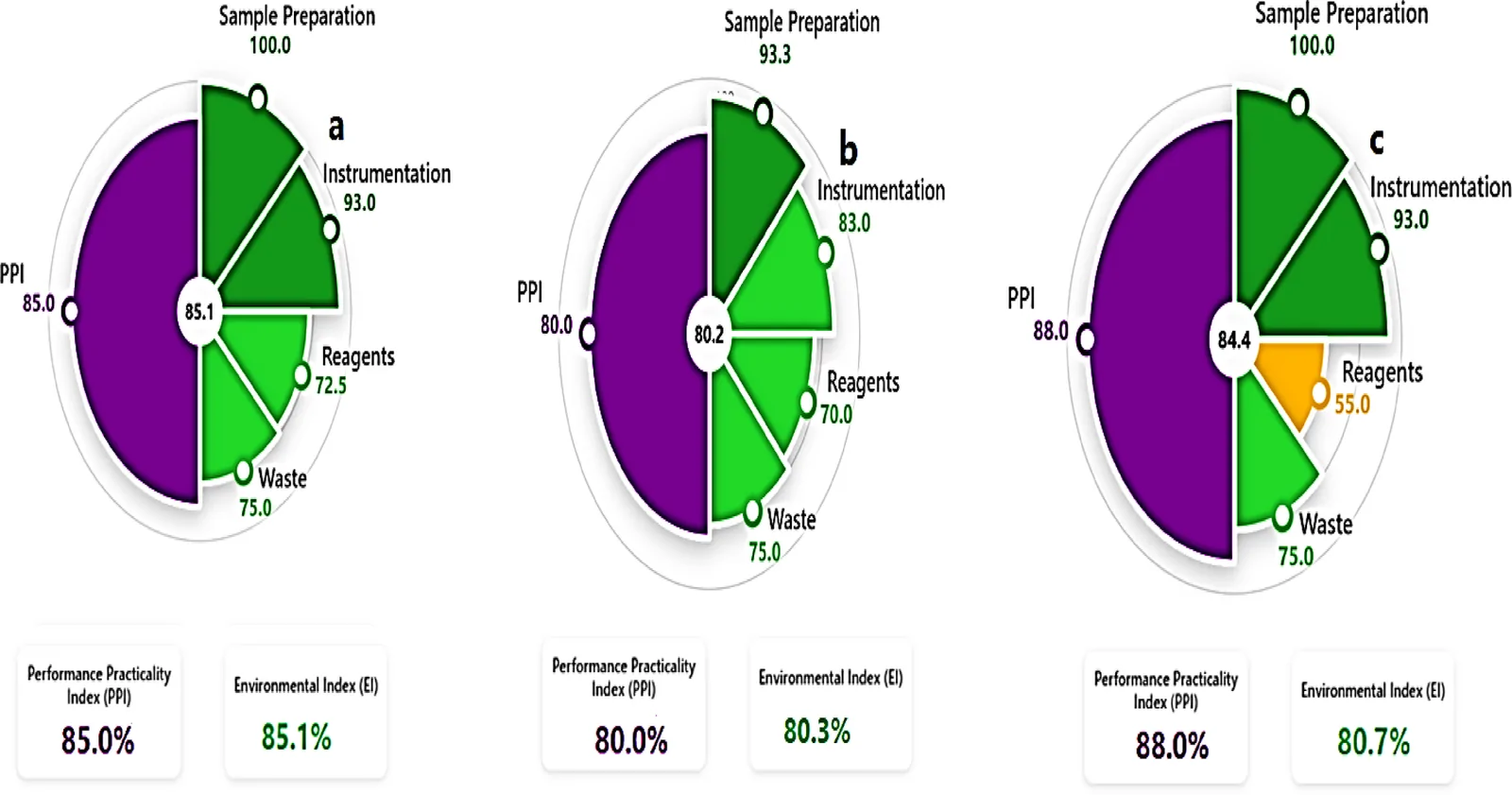
Graphical representation of EPPI index for (**a**) spectrophotometric method, (**b**) RP-HPLC method and (**c**) reported fluorometric method.

**Fig. 9:**
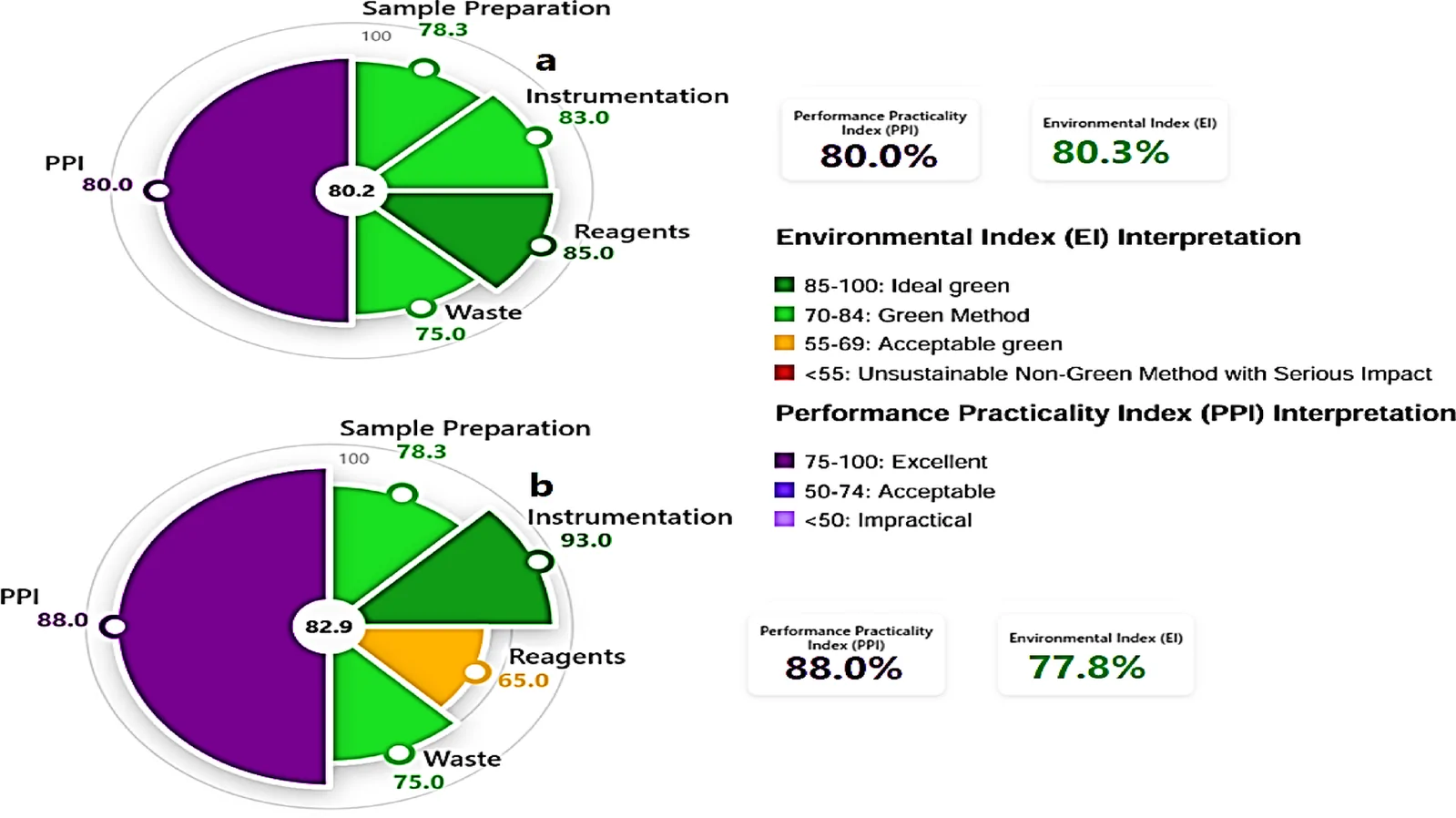
Graphical representation of EPPI index for (**a**) the proposed RP-HPLC, and (**b**) the reported fluorometric method for biological samples.

Greenness assessment has long been a crucial part of analytical method development^16–19^. The sustainability and overall analytical effectiveness of the developed methods were assessed using the EPPI framework. In EPPI, the first step includes evaluation of EI score which judges’ preparation of sample, instrument, used solvents and produced waste. For spectrophotometric and spectrofluorometric methods, no need for sample filtration and less energy is consumed than chromatographic method. For the used solvents and reagents, the reported method used sodium dodecyl sulphate in addition to acetonitrile as solvents which highly affects the EI score. So, spectrophotometric methods are ideal green method with minimal impact (EI = 85–100), while the RP-HPLC method and the reported one are considered only green (Fig. 8). The second step depends on evaluation of PPI score which judges the practicality and performance of the method according to sensitivity, cost, time and validation of the method. The larger the PPI score, the more will be the practicality and higher performance of the method. The three methods have PPI scores ≥ 75 which means that all the methods are of excellent practicality, high applicability and efficiency. The reported method has a PPI score larger than both proposed methods due to the high sensitivity of the fluorometric method in nanograms. Also, spectrophotometric and spectrofluorometric methods have larger PPI values than RP-HPLC due to short analysis time that leads to high throughput of these methods. So, the total EPPI score of spectrophotometric methods is larger than that of the reported method which is in turn larger than that of RP-HPLC one. But all methods have EPPI score (on the center of graphical representation) larger than 75 which reveals that all methods are green, practical and efficient methods. As for the biological fluid samples, The EPPI values of the proposed RP-HPLC and reported method still larger than 75 that means that they are practical, efficient and eco-friendly methods (Fig. 9). The EPPI scores for biological fluids differ on sample preparation which includes extraction steps and complex biological matrices. But RP-HPLC method uses a small volume of mobile phases for reconstitution of biological sample (0.5-mL) which is less than the solvent volume used in reported method. So, the EI value of the RP-HPLC method is more than that of the reported one. It is worth- mentioning that the PPI score of RP-HPLC is lower than reported method because of the high sensitivity of the spectrofluorometric method as shown in Fig. 9.

**Table 7:** Comparison between the proposed methods and reported one.

Parameters	Proposed Spectrophotometric methods				Proposed HPLC method				Reported spectrofluorimetric method^10^	
	CPR		CLX		CPR		CLX		CPR	CLX
	Method I	Method II	Method I	Method II	DAD	FLD	DAD	FLD		
Wavelength	318 nm	280 nm	266 nm	254 nm	280 nm	λ_ex_ = 275 nm, λ_em_ = 445 nm	252 nm	λ_ex_ = 245 nm, λ_em_ = 382 nm	438 nm	364 nm
Linearity* range	10.0–60.0	3.0–20.0	5.0–30.0	2.0–20.0	1.0-350.0	0.2–100.0	2.0-400.0	0.5–200.0	5-200000	10-10000
LOQ*	2.12	0.68	0.85	0.67	0.94	0.15	1.37	0.27	0.72	1.77
LOD*	0.69	0.22	0.28	0.22	0.31	0.05	0.45	0.09	0.24	0.58
EPPI score	85.1				80.2				84.4	
EPPI score (biological analysis)	-------------				80.2				82.9	
Advantages	Method I provides a simple, rapid, and economical approach suitable for routine quality control, whereas Method II offers higher sensitivity and selectivity, particularly in the presence of spectral overlap.				It has superior separation and selectivity, reduced effect of biological matrix, multi-component analysis in the same run and the variety in detection system.				It has high sensitivity, low LOQ and LOD and wide linearity range.	

*Unit of linearity range, LOQ and LOD is in µg/mL for proposed methods and in ng/mL for reported one.

## Conclusion

The present work demonstrates that combining spectrophotometric techniques with RP-HPLC analysis offers a powerful and versatile analytical strategy for the simultaneous determination of CLX and CPR in laboratory-prepared tablets and in rat plasma in ratio 1:10. The developed spectrophotometric methods provide a rapid, cost-effective, and environmentally considerate approach that is well suited for routine quality-control laboratories, particularly where simplicity and high sample throughput are required. Their ability to resolve spectral overlap through measurement of isosbestic point and derivative ratio processing highlights their practicality without sacrificing analytical reliability. In parallel, the RP-HPLC method complements these approaches by delivering superior sensitivity and selectivity, making it valuable especially for complex matrices such as pharmaceutical formulations and biological samples. The use of a RP system ensures efficient separation, reproducible quantification, and minimal matrix interference. Such factors are critical requirements for pharmacokinetic and bioanalytical applications. The integration of both analytical methods allows each method to compensate for the limitations of the other, offering a balanced framework that meets diverse analytical needs. Furthermore, the favorable environmental and practical outcomes assessed through the EPPI model emphasize that high analytical performance can be achieved without compromising sustainability. Collectively, these methods represent a forward-looking analytical tool that supports reliable drug monitoring, efficient quality control, and greener pharmaceutical analysis.

## Data Availability

Data will available upon request.

## Abbreviations

ALS: Amyotrophic lateral sclerosis
CLX: Celecoxib
CPR: Ciprofloxacin
DAD: Diode array detector
FLD: Fluorescence detector
GSP: Green shipping practices
GAC: Green analytical chemistry
WAC: White analytical chemistry
EPPI: Environmental performance and practicality index
RP-HPLC: Reverse-phase high performance liquid chromatography
ACN: Acetonitrile
FDR: First derivative ratio
Min: Minute
IS: Internal standard
LQC: Low quality control
MQC: Medium quality control
HCQ: High quality control
SSP: System suitability parameters
k': Capacity factor
t_R_: Retention time
R_s_: Resolution
ɑ: Selectivity factor
N: Number of theoretical plates
T: Tailing factor
ICH: International Conference on Harmonization
S_a_: Standard deviations of the intercept
S_b_: Standard deviations of the Slope
r: Correlation coefficient
S_y/x_: Standard deviations of residuals
LOD: Limit of detection
LOQ: Limit of quantification
SD: Standard deviation
%R: Percentage recovery
%RSD: Percentage relative standard deviation
%E_r_: Percentage relative error
FDA: Food and Drug Administration
EI: Enviromental index
